# Relative Performance of Machine Learning and Linear Regression in Predicting Quality of Life and Academic Performance of School Children in Norway: Data Analysis of a Quasi-Experimental Study

**DOI:** 10.2196/22021

**Published:** 2021-07-16

**Authors:** Robert Froud, Solveig Hakestad Hansen, Hans Kristian Ruud, Jonathan Foss, Leila Ferguson, Per Morten Fredriksen

**Affiliations:** 1 School of Health Sciences Kristiania University College Oslo Norway; 2 Clinical Trials Unit Warwick Medical School University of Warwick Coventry United Kingdom; 3 Department of Computer Science University of Warwick Coventry United Kingdom

**Keywords:** modelling, linear regression, machine learning, artificial intelligence, quality of life, academic performance, continuous/quasi-continuous health outcomes

## Abstract

**Background:**

Machine learning techniques are increasingly being applied in health research. It is not clear how useful these approaches are for modeling continuous outcomes. Child quality of life is associated with parental socioeconomic status and physical activity and may be associated with aerobic fitness and strength. It is unclear whether diet or academic performance is associated with quality of life.

**Objective:**

The purpose of this study was to compare the predictive performance of machine learning techniques with that of linear regression in examining the extent to which continuous outcomes (physical activity, aerobic fitness, muscular strength, diet, and parental education) are predictive of academic performance and quality of life and whether academic performance and quality of life are associated.

**Methods:**

We modeled data from children attending 9 schools in a quasi-experimental study. We split data randomly into training and validation sets. Curvilinear, nonlinear, and heteroscedastic variables were simulated to examine the performance of machine learning techniques compared to that of linear models, with and without imputation.

**Results:**

We included data for 1711 children. Regression models explained 24% of academic performance variance in the real complete-case validation set, and up to 15% in quality of life. While machine learning techniques explained high proportions of variance in training sets, in validation, machine learning techniques explained approximately 0% of academic performance and 3% to 8% of quality of life. With imputation, machine learning techniques improved to 15% for academic performance. Machine learning outperformed regression for simulated nonlinear and heteroscedastic variables. The best predictors of academic performance in adjusted models were the child’s mother having a master-level education (*P*<.001; β=1.98, 95% CI 0.25 to 3.71), increased television and computer use (*P*=.03; β=1.19, 95% CI 0.25 to 3.71), and dichotomized self-reported exercise (*P*=.001; β=2.47, 95% CI 1.08 to 3.87). For quality of life, self-reported exercise (*P*<.001; β=1.09, 95% CI 0.53 to 1.66) and increased television and computer use (*P*=.002; β=−0.95, 95% CI −1.55 to −0.36) were the best predictors. Adjusted academic performance was associated with quality of life (*P*=.02; β=0.12, 95% CI 0.02 to 0.22).

**Conclusions:**

Linear regression was less prone to overfitting and outperformed commonly used machine learning techniques. Imputation improved the performance of machine learning, but not sufficiently to outperform regression. Machine learning techniques outperformed linear regression for modeling nonlinear and heteroscedastic relationships and may be of use in such cases. Regression with splines performed almost as well in nonlinear modeling. Lifestyle variables, including physical exercise, television and computer use, and parental education are predictive of academic performance or quality of life. Academic performance is associated with quality of life after adjusting for lifestyle variables and may offer another promising intervention target to improve quality of life in children.

## Introduction

In trials and quasi-experimental designs, reported sample sizes range from less than 100 to several thousand [1]. Linear regression approaches are widely used for modeling continuous outcome data in such studies [2]. Processor advancements, data abundance, and routine data collection have cultivated a general rise in popularity of artificial intelligence or machine learning techniques. In contrast to regression, the use of machine learning techniques requires making fewer assumptions about data structure [3]. Machine learning techniques have been used extensively in areas such as biomedicine and, to a lesser extent, in areas such as chronic disease, pain, psychology, and sociology, where data have not typically been available in such abundance [4-6]. Machine learning techniques have yielded useful health classification models [7,8]. Numerous comparisons exist between machine learning techniques and traditional logistic or multinomial logit regression, demonstrating that approaches can yield similar performance and highlighting a risk of overfitting in machine learning techniques [9]. However, few comparisons exist between machine learning techniques and linear regression for continuous outcomes in health data sets, and where such comparisons have been made, sample sizes have been small [10,11].

Quality of life is an important health outcome in trials [2,12]. Child quality of life is associated with parental socioeconomic status and activity levels [13-16]. Diet is associated with child mental health, but the nature of the relationship between diet and child quality of life is less clear [17,18]. It has been suggested that aerobic fitness and muscular strength are positively associated with child quality of life [13,19]. The extent to which academic performance and quality of life are associated is also unclear. Known predictors of academic performance include parental socioeconomic status, child IQ, and activity levels, and there is some evidence of association with diet [20-22]. Thus, any relationship between quality of life and academic performance may be confounded by common associations with socioeconomic status, activity, and diet. Our aims were to examine the performance of linear regression and common machine learning techniques; the extent to which lifestyle variables (including physical activity, aerobic fitness, muscular strength, diet) and parental education are predictive of academic performance and quality of life; and the association between academic performance and quality of life, after adjusting for confounding variables, using a relatively large data set with continuous health outcomes.

## Methods

### Data Set

We used data from fifth-year students attending 9 schools in Norway between 2015 and 2019, within the Health Oriented Pedagogical Project (HOPP), which is an ongoing quasi-experimental study (ClinicalTrials.gov; NCT02495714) in which data up to 2019 were captured [23]. Schools were allocated to receive intervention (n=7) or usual curriculum (n=2). In intervention schools, child activity was increased by 225 minutes per week and an activity-based learning component (emphasizing mathematics and language studies, including English) was undertaken during the physical activity [23]. Both parent and child quality of life was measured using the Norwegian version of the Inventory of Life Quality [24]. The Norwegian Inventory of Life Quality has good internal consistency (normative 11- to 12-year-old children: Cronbach α=.82; parents: Cronbach α=.80) and good test–retest reliability in Norwegian children (normative 11- to 14-year-old children: intraclass correlation coefficient 0.86) [25]. In parents, test–retest has been reported as satisfactory, although we found no reports of a published intraclass correlation coefficient [25]. The Inventory of Life Quality spans domains of perceived school performance, family relations, peer relations, autonomy in play, physical health, mental health, and global assessment of well-being and uses a measurement scale of 0 to 100, where higher scores indicate greater quality of life. Academic performance was measured using the Norwegian Directorate for Education and Training’s compulsory National Academic Tests for fifth year students. We had access to reading, mathematics, and English test results; each academic subject was measured on a quasi-continuous scale (ranging from 0 to 100). Because we were interested in general academic performance, we used the average of these tests [26].

Physical activity level (defined by movement counts per minute: sedentary 0-99, light 100-1999, moderate 2000-4999, and hard or vigorous ≥5000 [13], while a monitor was worn between 8 hours and 6 days), percentage of time spent at each activity level, and average moderate-to-vigorous physical activity level (the sum of the minutes spent in moderate-to-vigorous activity divided by the number of valid monitored days) [27]; weight; height; blood pressure; waist circumference; muscle mass; percentage body fat; hand strength; aerobic fitness (Andersen intermittent running test [28]); executive functions (Stroop test [29,30]); parental education (university education or not; masters level or above or not); and lifestyle (self-reported diet, physical activity, and health questions from the Ungkost-2000 questionnaire [31]) data were included as predictor variables. Where there were missing observations in year 5 Ungkost variables, we carried forward observations from the same pupils in year 4.

### Modeling Approaches

We split the data set randomly into training (70%) and validation (30%) sets in order to train models and subsequently evaluate performance. We expected missing data (approximately 20% overall, with few variables >50%). Full imputation may often be performed with machine learning techniques regardless of the extent of missing data or whether or not data are missing at random. We performed a sensitivity analysis using single-mean imputation for continuous predictor variables and mode for nonbinary or categorical predictors (stratified by school) under the assumption that observations were missing at random. We tested this assumption for variables in final models by fitting a dummy variable for variable missingness, examining effect on outcome using 2-tailed independent *t* tests. In addition, we simulated variables with no missing data. We first examined strengths and limitations of different approaches, modeling academic performance with worked examples, and then modeled child quality of life.

### Regression Modeling

We took a pragmatic approach to regression modeling that we judged to approximate best practice. In cases of high between-predictor correlations (ρ>0.75), we selected 1 variable for modeling. In the absence of strong clinical or theoretical indications, we chose the variable that explained the most variance. To enable comparisons to regression approaches in which individuals are clustered by site, we fitted linear mixed models with a random intercept by school. We also built nonhierarchical models, without this random effect, to compare adjusted *R*^2^ like-for-like with machine learning techniques (in which clustering was not nominated). To facilitate comparison of residual mean square error (RMSE), we standardized variables by subtracting the mean and dividing by the standard deviation, which is required by machine learning techniques. For curvilinear relationships, we explored fitting polynomial terms. In the case of truly nonlinear relationships—variables that are not well modeled with a single linear predictor (notwithstanding polynomial terms)—we fitted splines (ie*,* piecewise fitting of models) [32].

The diet and lifestyle variables from the Ungkost-2000 questionnaire have multiple quasi-continuous responses (eg*,* for sugared soda consumption, response options ranged from ‘Never/rarely’ and increased incrementally over 7-levels to a maximum that indicated >7 glasses per day). Where responses were normally distributed, we treated the variables as quasi-continuous. If distributions did not satisfy normality criteria, we dichotomized variables using a cut-point [33]. Variables with significant crude effects were considered for an adjusted model. We took a manual approach to model building, using a combination of the lowest Akaike Information Criterion and variables that we judged to be clinically or theoretically useful for outcome prediction [34]. When modeling academic performance, in order to facilitate performance comparisons with partly automated machine learning techniques, we did not favor modifiable exposures, but instead, favored those we judged would explain the most variance. For quality of life, we built 2 models: (1) optimized for prediction and (2) based on modifiable exposures. Models for sensitivity analyses with imputed data were built independently.

### Machine Learning Techniques

We evaluated the performance of 4 machine learning techniques (Table 1) [35,36]. We selected machine learning techniques that were able to be used with continuous outcome measures (and not only binary or categorical), appear commonly in health research literature, and we judged health researchers would find comparisons useful. It is beyond the scope of this paper to explain each technique in detail; however, overviews are provided in Table 1.

Variables that it did not make sense to include were removed (eg*,* age, since participants were from the same school year). We set each approach to start with a null model and successively added variables that provided the best improvement, measured by RMSE in cross-validation [35]. We only selected tuning characteristics, such as the optimum value of *k* in k-nearest neighbor models, or optimum decay and threshold activation levels in neural network models, after graphical assessment. For machine learning techniques, we did not dichotomize nonnormal diet and lifestyle variables, since machine learning techniques are not sensitive to normality.

**Table 1:** Machine learning techniques that were evaluated in this study.

Algorithm	Description
k*-*Nearest neighbors	A classification technique that assigns class or predicts a continuous value based on the classes or values of *k* nearest neighbors.
Neural network	A technique in which artificial neuron cores are connected with *n* input channels, inputs are weighted and summed, and the output (if above an activation threshold) feeds into another neuron in a deeper hidden layer. This deeper neuron receives multiple inputs from each neuron in the layer above, and communicates output with either another hidden layer, or an output layer. Synaptic weights in this structure are determined by back propagation, based on error, until convergence is reached.
Random forest	An iteratively grown set of decision trees, where each tree outputs outcome means, with branches split by variable characteristics, and where each tree is formed from randomly bootstrapping data, with averages taken from all trees.
Support vector machine	A technique that minimizes error to individualize a hyperplane.

### Simulations

We simulated data to explore types of relationship that were not present within our real data, but which we reasoned, may perform better with either regression or machine learning techniques. We simulated, without missing data, (1) a variable with a quadratic relationship with academic performance; (2) a variable with a true nonlinear relationship with academic performance; and (3) a variable with marked heteroscedasticity (ie*,* changing variance) with respect to academic performance (we acknowledge this is a technical violation of regression; therefore, we recorded *R^2^* and RMSE rather than standard error terms). We permitted slight heteroscedasticities to remain in the first 2 simulations to approximate limits of real-life pragmatic decisions. We expected curvilinear simulation to favor regression, since we reasoned it would be modeled well with polynomial terms; nonlinear simulation to favor machine learning techniques, or linear regression with splines, since truly nonlinear relationships are not conducive to modeling by a single linear predictor; and heteroscedastic simulation to favor machine learning techniques, since modeling is not derived using minimum squared error, which in the presence of heteroscedasticity would no longer be the best estimator.

### Performance Comparisons and Using Worked Examples for Modeling Quality of Life

To compare performance, we calculated RMSE and *R^2^* using predicted observations from training sets and observed observations from validation sets (Multimedia Appendix 1). Informed by findings from modeling academic performance, we judged the most appropriate modeling technique for quality of life, and to confirm that we had made the correct choice, we compared the performance of the approaches that we selected with those that we did not select.

To aid interpretation of adjusted regression model outputs for those unfamiliar with the outcome scales, we calculated Cohen *d* for our judgements of clinically intuitive predictor magnitudes, by outcome variable; where *d* may be interpreted by thresholds of small (0.2), medium (0.5), and large (0.8) effects [37].

All analyses were performed using Stata (version 15.1; StataCorp LLC) and R (version 3.6; R Foundation for Statistical Computing). The HOPP project received approval from the Norwegian Regional Ethical Committee (2014/2064/REK south-east), and parents of all children provided written informed consent for their child’s participation.

## Results

### Overview

Data comprised outcomes from 1711 year 5 (11- and 12-year-old) children (Tables S1 and S2 in Multimedia Appendix 1), of whom 1368 (80.0%) had completed National Test outcomes and 1560 (91.6%) had completed quality of life outcomes. Missing data ranged from 4% to 81%, by variable. Our training and validation data sets had data from 1205 and 506 children, respectively.

### Academic Performance and Simulated Data

**Figure 1:**
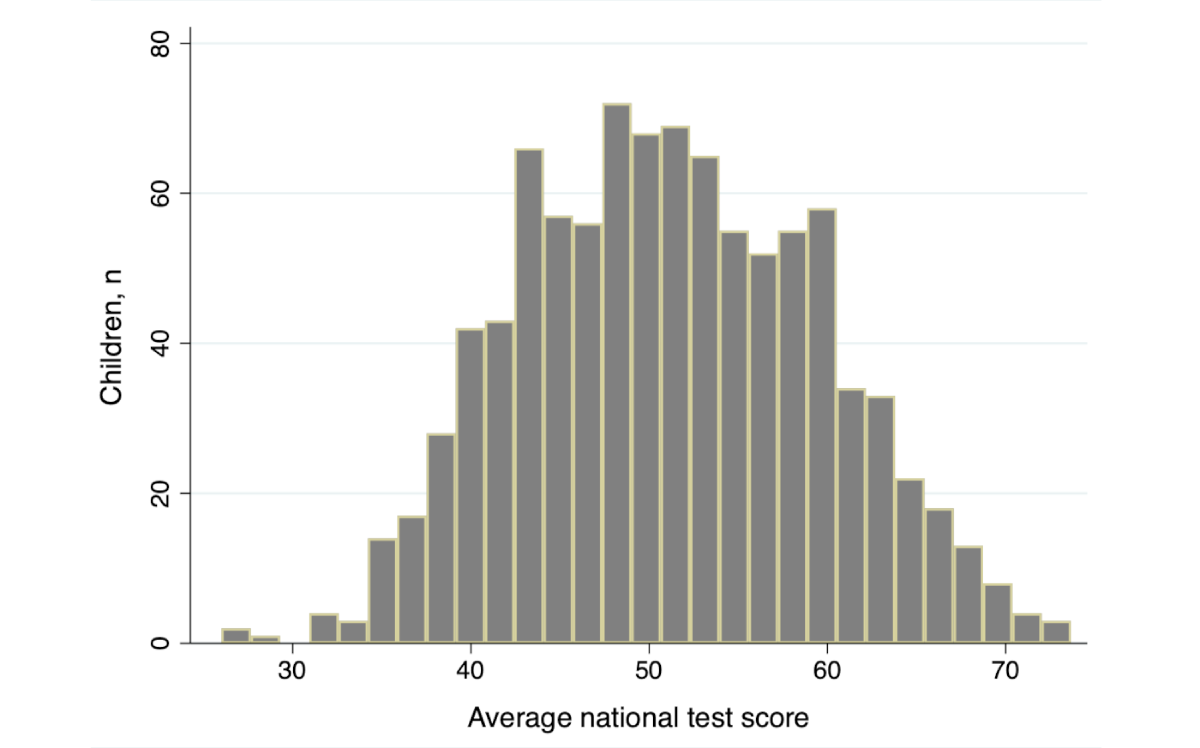
Histogram of average national test scores.

Academic performance was approximately normally distributed (Figure 1). From crudely modeled academic performance variables (Table S3 in Multimedia Appendix 1), we selected 7 variables for modeling (Table 2). We noted that after adjustment, dietary variables either explained too little variance or had too few observations for us to select for inclusion. Machine learning techniques did retain some dietary variables (Table S4 in Multimedia Appendix 1).

In real complete-case data, nonhierarchical and mixed models explained approximately 30% of the variance in the training set and 22% to 24% of the variance in the validation set (Table 3). Model residuals were normally distributed. Machine learning models explained between 13% and 63% of the variance in the training set and approximately 0% of the variance in validation (Table 3).

**Table 2:** Adjusted effects in selected mixed regression model for predicting academic performance.

Variable	*β* (95% CI)	n	*P* value
Stroop test congruent (milliseconds)	−0.0037 (−0.0047 to −0.0027)	384	<.001
Effect of master-level education for father	1.59 (−0.06 to 3.25)	384	.06
Effect of master-level education for mother	1.98 (0.25 to 3.71)	384	<.001
Average hand strength (kilograms)	0.21 (0.08 to 0.34)	384	.001
Hours of physical activity (self-reported; dichotomized)	2.47 (1.08 to 3.87)	384	.001
Effect of mother having higher education	1.82 (0.07 to 3.57)	384	.04
Hours of television per week (self-reported; 7-level quasi-continuous)	1.19 (0.25 to 3.71)	384	.03

**Table 3:** Performance indicators in real data and real data augmented with simulated data (quadratic, nonlinear, or heteroscedastic) for academic performance.

Model			Training (n=962)							Validation (n=406)				
		RMSE^a^		*R*^2^ value^b^		n		RMSE			*R*^2^ value^b^		n	
**Nonhierarchical linear model**			0.81		0.30		384		0.85			0.22		163
	Quadratic	0.45		0.78		384		0.40			0.83		163	
	Nonlinear	0.55		0.68		384		0.53			0.70		163	
	Heteroscedastic	0.53		0.70		384		0.61			0.61		163	
**Mixed model**			0.83		0.30		384		0.86			0.24		163
	Quadratic	0.46		0.79		384		0.39			0.84		163	
	Nonlinear	0.56		0.68		384		0.53			0.72		163	
	Heteroscedastic	0.54		0.70		384		0.62			0.62		163	
**Regression with splines**			—^c^		—		—		—			—		—
	Nonlinear	0.41		0.82		384		0.39			0.84		163	
**Random forest**			0.61		0.62		121		0.95			−0.02		63
	Quadratic	0.32		0.91		121		0.51			0.75		63	
	Nonlinear	0.36		0.89		121		0.57			0.64		63	
	Heteroscedastic	0.34		0.89		121		0.67			0.53		63	
**Support vector machine**			0.55		0.63		116		0.89			−0.05		58
	Quadratic	0.33		0.87		116		0.53			0.62		58	
	Nonlinear	0.46		0.77		116		0.77			0.18		58	
	Heteroscedastic	0.35		0.85		116		0.62			0.52		58	
**k-Nearest neighbors**			0.90		0.13		133		1.02			−0.01		66
	Quadratic	0.37		0.84		133		0.48			0.75		66	
	Nonlinear	0.41		0.81		133		0.48			0.75		66	
	Heteroscedastic	0.43		0.79		133		0.61			0.60		66	
**Neural network**			0.73		0.35		124		1.03			−0.02		66
	Quadratic	0.38		0.82		124		0.40			0.85		66	
	Nonlinear	0.41		0.79		124		0.46			0.79		66	
	Heteroscedastic	0.43		0.77		124		0.70			0.53		66	

^a^RMSE: residual mean square error.

^b^Unlike unadjusted *R^2^*, it is possible for adjusted *R^2^* values to be negative.

^c^Not performed.

**Figure 2:**
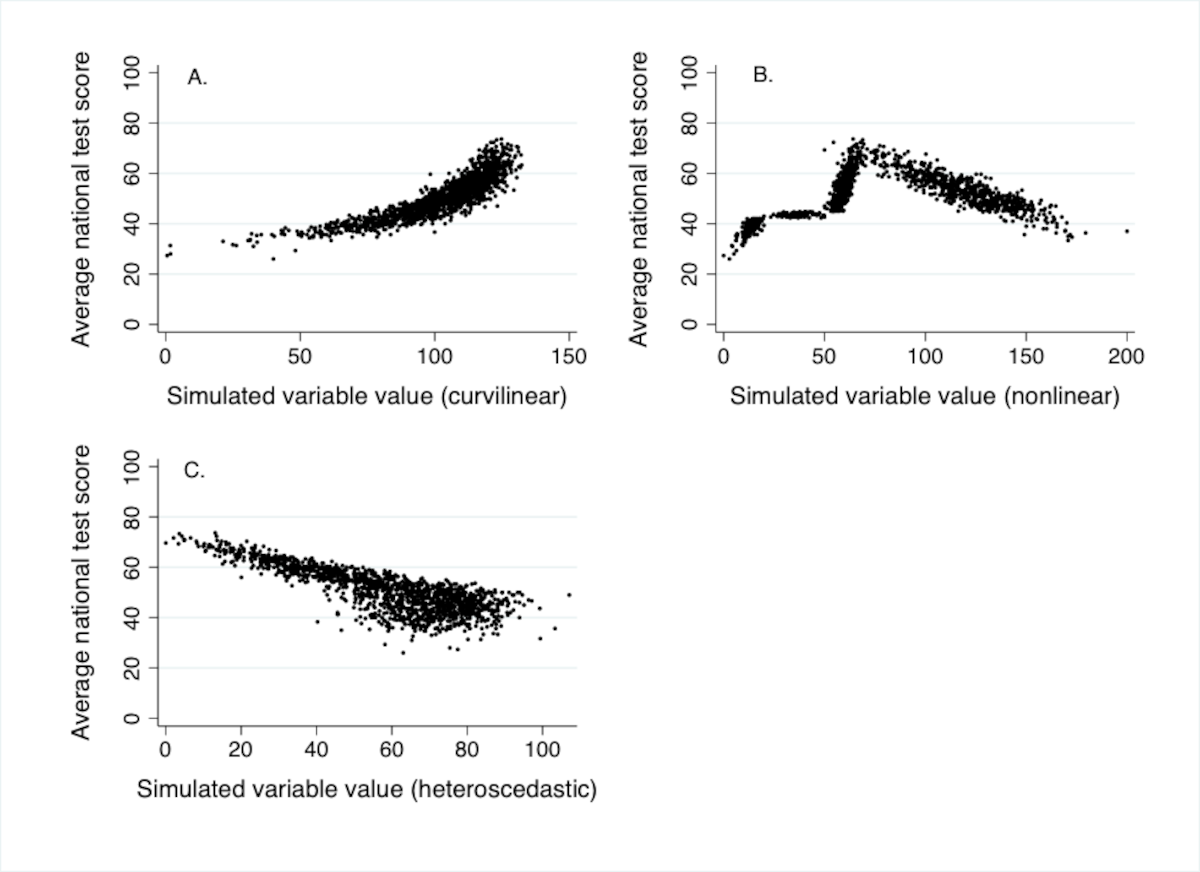
Scatter plots of average national test score and simulated (A) curvilinear, (B) nonlinear, and (C) heteroscedastic variables.

Figure 2 shows scatter plots of academic performance and simulated variables. All had strong effects in regression models when modeled as quadratic, quadratic, and linear. Adding a simulated quadratic variable to crude regression models explained approximately 79% of the variance in the training set and 82% to 83% of the variance in the validation (Table 4). Corresponding machine learning models explained 80% to 94% of the variance in the training set and 78% to 83% of the variance in the validation set, with support vector machine and neural network performing best. The nonlinear simulation was the only one with a variable that had a nonlinear relationship with academic performance, and we fitted 4 splines. Regression with splines explained 83% of the variance in the training set and 85% of the variance in the validation set. Corresponding machine learning models explained 81% to 94% of variance in the training set and 81% to 86% of the variance in the validation set, with neural network performing best. Adding a simulated heteroscedastic variable to crude regression models explained 64% of variance in the training set and 62% of the variance in the validation set. Corresponding machine learning models explained 68% to 90% of the variance in the training set and 58% to 66% of the variance in the validation set, with neural network and support vector machine performing best.

Regression performed best for modeling real data augmented with simulations (Table 3). Regression with splines performed best when adding the nonlinear simulated variable. Table 5 shows machine learning performance improved after imputation; however, regression models outperformed machine learning. Regression models built using imputed data included 13 variables (Multimedia Appendix 1). Variables selected by machine learning techniques are shown in Table S4 in Multimedia Appendix 1. The missing at random assumption was widely acceptable, with 3 out of 35 variables selected for modeling (master’s education or above for mother, master’s education or above for father, and parent quality of life score) having an effect on academic performance.

**Table 4:** Crude performance of simulated variables.

Model		Training (n=962)			Validation (n=406)		
		RMSE^a^	*R*^2^ value	n	RMSE^a^	*R*^2^ value	n
**Nonhierarchical linear model**							
	Quadratic	0.45	0.79	962	0.43	0.82	406
	Nonlinear	0.56	0.68	962	0.58	0.68	406
	Heteroscedastic	0.59	0.64	962	0.63	0.62	406
**Mixed model**							
	Quadratic	0.45	0.79	962	0.43	0.83	406
	Nonlinear	0.57	0.68	962	0.58	0.68	406
	Heteroscedastic	0.59	0.64	962	0.63	0.62	406
**Regression with splines**							
	Nonlinear	0.41	0.83	962	0.39	0.85	406
**Random forest**							
	Quadratic	0.25	0.94	962	0.49	0.78	406
	Nonlinear	0.24	0.94	962	0.45	0.81	406
	Heteroscedastic	0.32	0.90	962	0.66	0.58	406
**Support vector machine**							
	Quadratic	0.44	0.80	962	0.42	0.83	406
	Nonlinear	0.44	0.81	962	0.43	0.82	406
	Heteroscedastic	0.57	0.68	962	0.61	0.66	406
**k-Nearest neighbors**							
	Quadratic	0.40	0.84	962	0.45	0.81	406
	Nonlinear	0.34	0.88	962	0.43	0.82	406
	Heteroscedastic	0.49	0.76	962	0.65	0.60	406
**Neural network**							
	Quadratic	0.44	0.80	962	0.42	0.83	406
	Nonlinear	0.40	0.84	962	0.38	0.86	406
	Heteroscedastic	0.56	0.68	962	0.59	0.66	406

^a^RMSE: residual mean square error.

**Table 5:** Performance indicators for academic performance in sensitivity analyses (single-mean imputation).

Model	Training (n=962)				Validation (n=406)		
	RMSE^a^	*R*^2^ value	n	RMSE^a^		*R*^2^ value	n
Nonhierarchical linear model	0.88	0.20	962	0.92		0.15	406
Mixed model	0.89	0.21	962	0.92		0.18	406
Random forest	0.76	0.48	962	0.94		0.14	406
Support vector machine	0.82	0.32	962	0.95		0.12	406
k-Nearest neighbors	0.89	0.20	962	0.86		0.12	406
Neural network	0.90	0.18	962	0.97		0.09	406

^a^RMSE: residual mean square error.

### Quality of Life

**Figure 3:**
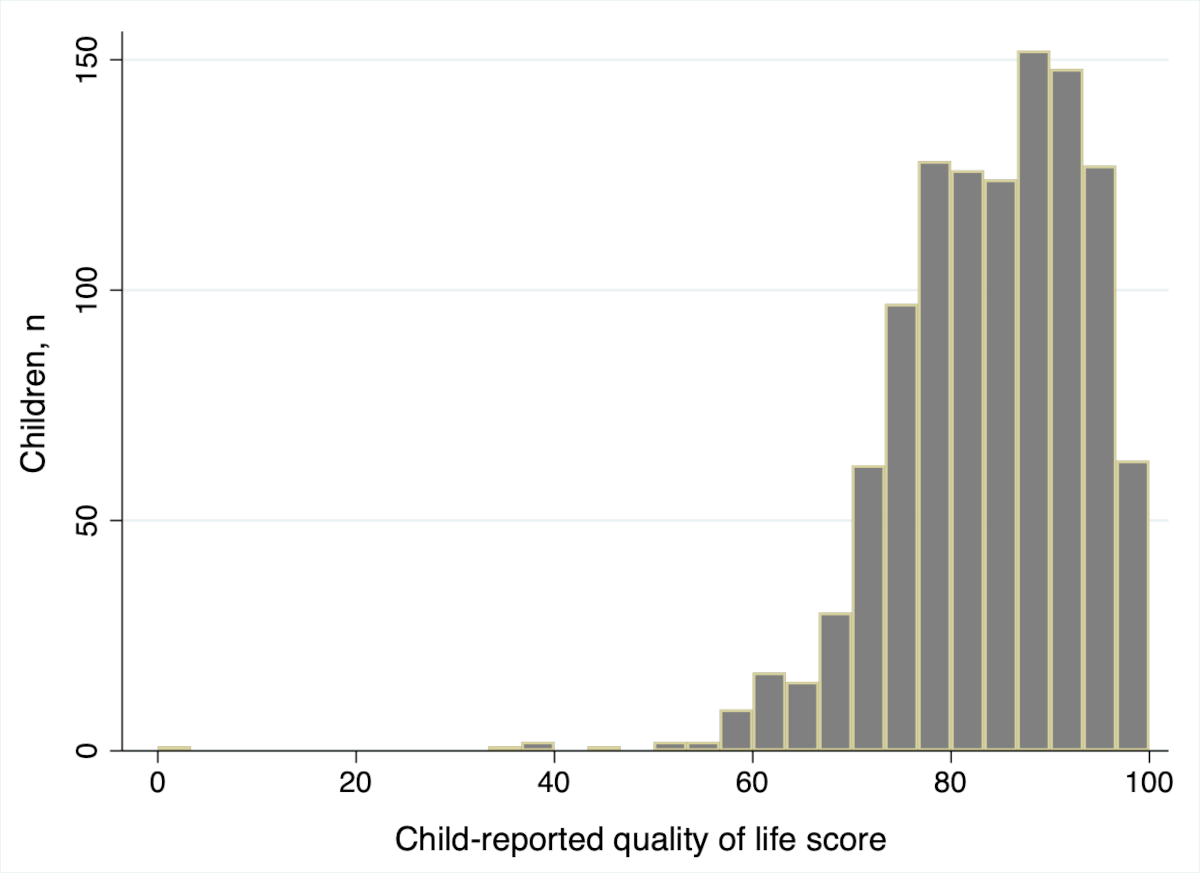
Histogram of child-reported quality of life scores.

Despite a ceiling effect, we judged the distribution of child-reported quality of life (Figure 3) to be within limits of tolerance for untransformed parametric modeling (and we confirmed there was a normal distribution of residuals postmodeling). Since visual inspection revealed no nonlinear relationships, and only very slight heteroscedasticity at times, we judged regression modeling would perform best. We dichotomized 1 diet variable (fish oil consumption) based on crude effects (Table S5 in Multimedia Appendix 1). We selected a parsimonious 3-variable model (Regression model 1) on the basis of raw performance (Table S6 in Multimedia Appendix 1) and a second 4-predictor model (Regression model 2) using only variables with a high number of observations and representing modifiable risk factors (Table 6). When added, academic performance had a significant association with quality of life (*P*=.02), with an adjusted effect of 0.12 (95% CI 0.02 to 0.22). We did not include academic performance in our comparative model because it reduced observations and led to lower training *R*^2^ values. Two of the machine learning techniques retained academic performance and several diet variables in addition to fish oil (Table S4 in Multimedia Appendix 1).

**Table 6:** Adjusted effects of with modifiable risk factors in mixed regression model for predicting quality of life.

Variable	β (95% CI)	n	*P* value
Frequency of physical activity (7-level quasi-continuous)	1.09 (0.53 to 1.66)	676	<.001
Hours of television per week (self-reported; 7-level quasi-continuous)	−0.95 (−1.55 to −0.36)	676	.002
Hard exercise (minutes)	0.02 (0.002 to 0.03)	676	.008
Percentage of time in moderate exercise	0.29 (0.002 to 0.59)	676	.048

Our parsimonious 3-variable mixed model explained 12% of variance in the training set and 15% of the variance in the validation set. Machine learning techniques retained more observations than the first regression model due to our selection of the fish oil variable, which had fewer observations (Table 7). Our second 4-predictor model explained 8% of the variance in the training set and 6% to 7% of the variance in the validation set. This was outperformed by support vector machine; however, our second regression model retained more observations and had been limited by us to modifiable risk factors.

**Table 7:** Performance indicators by modeling approach for quality of life.

Model	Training (n=1107)				Validation (n=453)		
	RMSE^a^	*R*^2^ value	n	RMSE^a^		*R*^2^ value	n
Regression model 1	0.89	0.11	293	0.85		0.13	111
Mixed model 1	0.89	0.12	293	0.85		0.15	111
Regression model 2	0.91	0.08	676	0.95		0.06	275
Mixed model 2	0.91	0.08	676	0.96		0.07	275
Random forest	0.66	0.74	481	0.89		0.03	190
Support vector machine	0.85	0.14	524	0.97		0.08	208
k-Nearest neighbors	0.78	0.33	295	0.97		0.08	117
Neural network	0.80	0.28	319	0.99		0.07	123

^a^RMSE: residual mean square error.

Table 8 shows the results from imputed sensitivity analyses. Regression models included 8 variables (Multimedia Appendix 1). The variables selected by the machine learning techniques are shown in Table S4 in Multimedia Appendix 1. The missing at random assumption was mostly acceptable, with 5 out of the 17 variables selected for modeling (hard exercise, percentage of time in moderate and light exercise, parent quality of life score, and master’s education for father) having an effect on quality of life.

**Table 8:** Performance indicators by modeling approach for quality of life in sensitivity analysis (single-mean imputation).

Model	Training (n=1107)			Validation (n=453)		
	RMSE^a^	*R*^2^ value	n	RMSE^a^	*R*^2^ value	n
Regression model	0.95	.09	1107	0.93	.13	453
Mixed model	0.95	.09	1107	0.93	.14	453
Random forest	0.80	.59	1107	0.96	.05	453
Support vector machine	0.92	.17	1107	0.96	.07	453
k-Nearest neighbors	0.94	.12	1107	0.96	.06	453
Neural network	0.96	.09	1107	0.97	.05	453

^a^RMSE: residual mean square error.

## Discussion

### Principal Results and Comparisons to Existing Research

In modeling continuous health outcomes in a data set containing some missing data, linear regression was less prone to overfitting, retained more observations, and outperformed common machine learning techniques. In validation, regression explained approximately one-quarter of the variance in academic performance and up to 15% of the variance in quality of life, using exercise, lifestyle, and parental education quality of life data. Imputation improved machine learning performance, but improvements were not sufficient to outperform regression. Machine learning techniques outperformed regression for modeling nonlinear and heteroscedastic simulations and may be of use when there are no missing data or imputation is plausible, and where complex nonlinearity or heteroscedasticity exists. However, regression with splines performed almost as well for nonlinear modeling.

Multiple comparisons exist between machine learning techniques and logistic regression, multiclass, and survival analysis models, which taken together suggest similar results and an increased risk of overfitting with machine learning techniques [9,38-44]. However, few comparisons exist between machine learning techniques and linear regression for continuous health outcome measures. Hoffman et al [10] compared linear regression and support vector machine to predict Oswestry Disability Index score after surgery and found an adjusted *R^2^* of 0.42 for linear regression and 0.93 from support vector machine in a sample of 20 individuals. We observed that *R^2^* for support vector machine in our academic performance training set was approximately twice those for linear regression. However, the same relationship is not borne out in validation, suggesting the high *R^2^* value in the primary data is an artefact of overfitting. Laitinen and Räsänen [45] compared a regression equation with neural network in a sample of 125 patients with congenital heart disease and found that neural network performed best. However, the neural network used study data alone, and thus, was likely subject to overfitting, while the regression equation was externally validated. Hayward et al [11], in 91 patients with pancreatic cancer, compared linear regression to several machine learning techniques, including decision trees, k-nearest neighbors, and neural network across a range of outcomes. They reported machine learning techniques and regression were comparable in 45 (35%) comparisons, machine learning techniques were superior in 33 (25%) comparisons, and machine learning techniques were inferior in 52 (40%) comparisons [11]. Our study uses more data than were used in prior work and more clearly demonstrates the superiority of linear regression for modeling continuous outcomes.

We found very strong evidence that reported physical activity, time recorded in vigorous exercise, and percentage of time spent in moderate exercise are positively associated with quality of life as continuous health outcomes in typical circumstances when adjusted for each of the other modeled variables. Associations between socioeconomic status, increased physical activity, and child quality of life are well established [13-15,46-48]. It has been suggested that the association may be explained via mechanisms involving affective response, increased self-efficacy, and improved mood-regulating neurotransmitter and endorphin release [14,49,50]. We found strong evidence that television and computer use is inversely proportional to quality of life. Increases of 1 use level (eg, going from 0 to 2 hours use per day), 100 minutes of vigorous exercise, or a 10% increase in exercise, are associated with small or small-to-medium (Multimedia Appendix 1) effects on quality of life. A systematic review [51] of physical activity and sedentary behavior on child quality of life found consistent evidence that watching television, using computers, or playing video games for more than 2 hours per day was significantly associated with lower child or adolescent quality of life. We found very strong evidence that parental assessment of child quality of life is associated with child quality of life assessment; this has been noted previously [25]. We found some evidence of association between academic performance and quality of life after adjustment; a 20-unit increase academic performance was associated with a small quality of life increase, and we are aware of no comparative work.

We found very strong evidence that reported physical activity, increased hand strength, mother having master’s education or above, and decreased Stroop time, are associated with increases in academic performance. We found some evidence that a mother having university education and increases in television and computer use, are associated with increased academic performance. Reporting exercise that causes a sweat for at least 2 hours per week, 10 kg greater hand strength, a mother having university or master’s education, increases of 1 television and computer use level, or a decreased Stroop time of 1 second were each associated with small or small-to-medium increases in academic performance. Socioeconomic status variables have been shown, in a meta-analysis [52] of 101,157 students, to be positively correlated with academic performance (with medium effect sizes), which is consistent with our findings. The role of socioeconomic status (ie, including parental education) may be explained by modified risk factors and health behaviors or self-concept [47,53]. Several mechanisms underlying a link between physical activity and academic performance have been suggested, which are thought to involve maintenance and facilitation of the plasticity of brain structures through altered neurogenesis and angiogenesis, enhanced central nervous system metabolism, and increased availability of growth factors [54-56]. An association between increasing physical activity and academic performance was demonstrated in a 2014 systematic review [57] of 215 studies. However, a 2019 systematic review [54] of 58 interventional studies of physical activity on cognitive performance, found only 10 out of 21 analyses (48%) in 5 high-quality studies demonstrated significant effects and found that the evidence was inconclusive. Furthermore, Singh et al [54] found only 15 of 25 analyses (60%) demonstrated academic performance benefits; stratification led to observation of strong evidence of a beneficial effect on math, but inconclusive evidence for language performance. Our own findings of an association between physical activity and general academic performance, come from using a composite outcome of reading, math, and English tests, and thus, future separate analyses may be of additional value.

Diet may affect both quality of life and academic performance via mechanisms related to the consumption of adequate micronutrients [17,58]. An association between healthy diet and the emotional functioning subscale of the Pediatric Quality of Life Inventory was demonstrated in a prospective study [18] of 3040 Australian adolescents (age 11 to 18 years). Our findings suggest small crude effects of diet across quality of life domains more generally. Decreased attendance, attention, and academic performance have been reported in undernourished children when compared to those reported in well-nourished children; fruit and vegetables, fat, and iron intake have been highlighted as having moderate effects in a study [58] of 5200 Canadian school children. A study [20] of 4245 Australian school-aged children (age 8-15 years) showed increased consumption of evening meal vegetables, breakfast consumption, and fruit are associated with higher spelling or writing scores, and increased sugar beverages are associated with lower scores. In our study, crude effects of increased sugared cordial consumption, sugar-free cordial, and pizza were associated with decreased academic performance generally but explained too little variance for us to select for inclusion in an adjusted model.

### Implications

The rising popularity of machine learning techniques is understandable given the general abundance of data and a need for fewer assumptions. Machine learning techniques may be useful simply by virtue of the amount of data available. However, in public health research and health services research, data are less abundant and often missing. When modeling continuous outcomes in such circumstances, machine learning techniques are likely to perform worse unless marked nonlinear or heteroscedastic relationships exist. We have shown that the tendency to overfit that is often demonstrated in binary and multiclass machine learning techniques is also a challenge when modeling continuous outcomes. Furthermore, an innate inability for parameter estimation hampers interpretation and may make machine learning techniques generally less useful. At the time of writing, machine learning techniques have made relatively little impact in public health research on COVID-19 (with either continuous or categorical outcomes) where there is a pressing and immediate need for good modeling. We find this unsurprising—in most cases, public health data have normal distributions, and marked nonlinearity is rare. In these cases, traditional regression methods use the most efficient estimators and will lead to better models.

Interventions aiming to improve activity levels in children may have a positive effect on both child quality of life and academic performance. The small association between academic performance and quality of life could follow satisfaction of achievement, although reversed causal direction, or residual confounding is plausible. In addition to increasing physical activity, new interventions to improve quality of life might target improvements in academic performance. Television and computer use is associated with decreases in quality of life but improvements in academic performance and these factors should be examined separately to clarify other promising intervention targets.

### Strengths, Limitations, and Recommendations for Future Research

We provide like-for-like comparisons between machine learning techniques and regression for modeling continuous health outcomes, with larger sample size than those used in previous research, and separate validation. Nevertheless, our work has limitations. We used an average of reading, math, and English tests as a proxy for academic performance. Not including subjects such as science may impair construct coverage of academic performance. Using single-mean imputation and last observation carried forward (in missing Ungkost variables) allowed us to avoid using multiple imputation (which is based on regression approaches) for data used in machine learning models (ie, to avoid mixing methods). However, multiple imputation provides better coverage than single-mean imputation, and last observation carried forward is known to be problematic [59]. It has been highlighted that the assumption of no change over (limited) time may hold in some contexts and can be better than ignoring missingness altogether [60]. In our case, we believed the assumption of no or limited change would be better than ignoring missingness completely or mixing methods when comparing regression approaches with machine learning techniques. There is a potential limitation regarding the validity and generalizability of results to 11- and 12-year-old children in the case of greater than assumed unobserved changes in missing Ungkost variables. With respect to single-mean imputation, our results showed that the missing at random assumption was not valid for some modeled variables. We believe that the applied techniques have been kept robust to imputation issues because results were in alignment with those from complete-case analyses; however, results derived from our imputed sensitivity analyses should be interpreted cautiously. Generalization of results to other countries should also be done with caution, since there may be baseline differences in activity and culture among Norwegian children. Finally, we focused on machine learning techniques that we judged to be the most common and which we thought researchers would find useful; we acknowledge that this is not a comprehensive comparison of regression with all possible machine learning techniques.

Future focus on comparisons to other machine learning techniques, separate analysis of academic performance components, and iteratively varying the size of the training set to explore how training set size affects overfitting will provide further useful knowledge. The Ungkost item on television and computer use combines 2 activities. We found large positive associations between the item and academic performance and a small negative association with quality of life. We suspect the positive associations may be grounded in computer use for education, and the negative associations may be grounded in uses for leisure. Separation of these exposures will provide clarity. Some machine learning techniques retained diet variables that we did not select for adjusted models. One strength of machine learning techniques may be an ability to detect mild and easily missed nonlinear relationships, which is worth further exploration.

### Conclusions

For modeling continuous health outcomes when some data are missing, linear regression is less prone to overfitting and outperforms common machine learning techniques. Imputation improves the performance of machine learning techniques, but improvements are not sufficient to outperform regression. Machine learning techniques outperform regression in modeling nonlinear and heteroscedastic relationships and may be of use in cases where imputation is sensible or there are no or few missing data. Otherwise regression is preferred. Regression with splines performs almost as well in nonlinear modeling. Lifestyle variables, including physical activity, television and computer use, muscular strength, and parental education were predictive of academic performance or quality of life explaining up to 24% and 15% of the variance in these outcomes, respectively. Targeting these areas in future interventions may help improve child quality of life and academic performance.

## Appendix

Multimedia Appendix 1 Supplementary tables and technical notes.

## Abbreviations

COVID-19: coronavirus disease 2019
HOPP: Health Oriented Pedagogical Project
RMSE: residual mean square error
