# Supplementary material for: Relative Performance of Machine Learning and Linear Regression in Predicting Quality of Life and Academic Performance of School Children in Norway: Data Analysis of a Quasi-Experimental Study
Source: J Med Internet Res. 2021 Jul 16;23(7):e22021. doi: 10.2196/22021 (PMC8325075; doi:10.2196/22021)
Supplement: Multimedia Appendix 1 [file jmir_v23i7e22021_app1.pdf]

Multimedia Appendix 1

Table S1 – Sample characteristics for continuous/quasi-continuous variables

Variable		*Training set (n=1205)*			*Validation set (n=506)*	
	n **(% missing)**	**Mean (SD)**	**Median (IQR)**	***n*** **(% missing)**	**Mean (SD)**	**Median (IQR)**
**Height (cm)**						
	1150 (4.6)	146 (6.8)	146 (9.5)	478 (5.7)	146.2 (7.0)	146 (9.5)
**Weight (kg)**						
	1143 (5.2)	38.6 (8.3)	37.2 (9)	477 (5.2)	39.1 (8.5)	37 (10.9)
**Waist circumference (cm)**						
	1122 (7.0)	66.7 (9.1)	65 (9)	464 (8.5)	67.0 (9.9)	64.3 (10)
**Systolic BP (mmHg)**						
	1092 (9.5)	109.4 (11.2)	109 (14)	451 (11.1)	108.4 (10.5)	108 (14)
**Diastolic BP**						
	1086 (10.0)	70.9 (8.0)	71 (10)	450 (11.2)	70.3 (8.8)	70 (10)
**Right Hand strength (kg)**						
	1119 (7.2)	16.7 (5.3)	16 (7)	458 (9.7)	16.82 (6.0)	16 (7)
**Left Hand strength (kg)**						
	1117 (7.4)	15.5 (5.2)	15 (7)	459 (9.5)	15.5 (5.5)	15 (6)
**Average hand strength (kg)**						
	1115 (7.6)	16.1 (5.1)	16 (7)	456 (10.1)	16.2 (5.6)	15.5 (6.5)
**Muscle mass (kg)**						
	1128 (6.5)	28.2 (4.5)	27.8 (5.4)	470 (7.3)	28.4 (4.5)	27.8 (6.1)
**Body fat (%)**						
	1128 (6.5)	22 (5.6)	20.9 (6.3)	470 (7.3)	22.3 (5.8)	21.4 (6.7)
**Fat mass (kg)**						
	1125 (6.7)	8.9 (4.5)	7.6 (4)	467 (7.9)	9.1 (4.4)	7.7 (4.4)
**Fat free mass (kg)**						
	1127 (6.6)	29.7 (4.7)	29.3 (5.7)	470 (7.3)	29.9 (4.8)	29.3 (6.2)
**Bone mass (kg)**						
	1129 (6.4)	1.6 (0.2)	1.6 (0.3)	470 (7.3)	1.6 (0.2)	1.6 (0.3)
**Protein mass (kg)**						
	1108 (8.1)	6.4 (1.0)	6.3 (1.3)	463 (8.7)	6.5 (1.0)	6.3 (1.4)
**Fitness test (meters)**						
	1045 (13.4)	943.3 (127.7)	942 (160)	444 (12.4)	940.1 (125.6)	950 (175)
**Stroop test congruent (ms)**						
	1015 (15.8)	1833.7 (794.3)	1632.9 (898.6)	411 (18.9)	1862.7 (903.3)	1631 (890.4)
**Stroop test incongruent (ms)**						
	1015 (15.8)	2204.5 (981.0)	1927.3 (1090.3)	411 (18.9)	2151.6 (1008.0)	1891.6 (993.9)
**Stroop control (ms)**						
	1015 (15.8)	1845.0 (808.3)	1647.9 (863.6)	411 (18.9)	1819.2 (852.6)	1605.7 (822.3)
**Sedentary exercise (minutes)**						
	1029 (14.7)	3372.2 (913.7)	3581.2 (954.2)	420 (17.2)	3387.0 (936.4)	3617.3 (1039.7)

**Light exercise (minutes)**						
	1029 (14.7)	1393.8 (398.8)	1438.7 (458.3)	420 (17.2)	1405.2 (387.8)	1466.3 (443.9)
**Moderate exercise (minutes)**						
	1029 (14.7)	445.6 (171.4)	438.2 (224.7)	420 (17.2)	445.3 (172.1)	434.3 (226.7)
**Hard exercise (minutes)**						
	1022 (15.3)	70.2 (60.2)	50.8 (66)	418 (17.6)	68.2 (60.8)	47.5 (62.4)
**Percentage of time sedentary**						
	1029 (14.7)	63.7 (6.1)	63.8 (8.2)	420 (17.2)	63.5 (5.9)	64.3 (7.7)
**Percentage of time in light exercise**						
	1029 (14.7)	26.5 (3.9)	26.3 (5.3)	420 (17.2)	26.7 (3.8)	26.6 (5.01)
**Percentage of time in moderate exercise**						
	1029 (14.7)	8.5 (2.6)	8.3 (3.3)	420 (17.2)	8.5 (2.7)	8.3 (3.4)
**Percentage of time in vigorous exercise**						
	1022 (15.3)	1.3 (1.1)	1.0 (1.2)	418 (17.6)	1.3 (1.1)	0.9 (1.2)
**Time in MVPA (minutes)**						
	1034 (14.3)	512.7 (212.7)	502.8 (290.0)	420 (17.2)	513.3 (213.3)	491 (284.1)
**Percentage of time in MVPA**						
	1034 (14.3)	9.8 (3.3)	9.4 (4.3)	420 (17.2)	9.8 (3.4)	9.4 (4.6)
**Average MVPA (minutes)**¶						
	1034 (14.3)	80.6 (27.8)	78.5 (37)	420 (17.2)	81.0 (29.1)	76.9 (38.3)
**Average steps (average cpm per day)**						
	1035 (14.2)	2.1 (0.6)	2 (0.7)	420 (17.2)	2.1 (0.6)	2 (0.7)
Average national test score						
	962 (20.2)	51.1 (8.4)	50.7 (12.7)	406 (19.9)	50.6 (8.6)	50 (12.3)
Child-rated child QoL (LQ100)						
	1107 (8.2)	84.4 (10.4)	85.7 (14.3)	453 (10.7)	84.9 (10.3)	87.5 (14.3)
**Parent-rated child QoL LQ100)**						
	702 (41.8)	87.4 (11.5)	89.3 (14.3)	273 (46.2)	88.0 (11.1)	89.3 (17.9)
**Frequency of eating lunch each week (8-level quasi-continuous)**						
	859 (28.7)	6.4 (2.2)	8 (3)	363 (28.3)	6.3 (2.3)	8 (3)
**Frequency of eating dinner each week (8-level quasi-continuous)**						

	859 (28.7)	6.5 (1.7)	6 (2)	363 (28.3)	6.6 (1.5)	6 (2)
**Frequency of eating supper each week (8-level quasi-continuous)**						
	859 (28.7)	3.6 (3.0)	1 (6)	363 (28.3)	3.9 (3.1)	2 (7)
**Frequency of eating packed lunch (6-level quasi-continuous)**						
	859 (28.7)	3.3 (2.3)	3 (5)	363 (28.3)	3.4 (2.3)	3 (5)
**Frequency of eating cafeteria lunch (7-level quasi-continuous)**						
	859 (28.7)	1.8 (1.5)	1 (1)	363 (28.3)	1.8 (1.5)	1 (1)
**Frequency of eating shop lunch (7-level quasi-continuous)**						
	859 (28.7)	2.4 (1.7)	1 (3)	363 (28.3)	2.3 (1.7)	1 (2)
**Frequency of whole milk consumption (7-level quasi-continuous)**						
	859 (28.7)	2.4 (1.8)	1 (3)	363 (28.3)	2.4 (1.8)	1 (3)
**Frequency of low-fat milk consumption (7-level quasi-continuous)**						
	859 (28.7)	2.1 (1.6)	1 (2)	363 (28.3)	2.2 (1.7)	1 (2)
**Frequency of extra-low-fat milk consumption (7-level quasi-continuous)**						
	859 (28.7)	2.5 (1.6)	2 (2)	363 (28.3)	2.5 (1.6)	2 (2)
**Frequency of skimmed milk consumption (7-level quasi-continuous)**						
	859 (28.7)	1.8 (1.2)	1 (1)	363 (28.3)	1.9 (1.3)	1 (1)
**Frequency of orange juice consumption (7-level quasi-continuous)**						
	859 (28.7)	2.1 (1.4)	2 (2)	363 (28.3)	2.1 (1.4)	2 (2)
**Frequency of sugared cordial consumption (7-level quasi-continuous)**						
	859 (28.7)	2.3 (1.1)	2 (2)	363 (28.3)	2.4 (1.2)	2 (2)
**Frequency of sugar-free cordial consumption (7-level quasi-continuous)**						
	859 (28.7)	1.8 (1.2)	1 (1)	363 (28.3)	1.8 (1.1)	1 (1)

**Frequency of sugared soda consumption (7-level quasi-continuous)**						
	859 (28.7)	1.9 (1.1)	2 (1)	363 (28.3)	1.9 (1.1)	2 (1)
**Frequency of sugar-free soda consumption (8-level quasi-continuous)**						
	859 (28.7)	2.3 (1.2)	2 (2)	363 (28.3)	2.5 (1.4)	2 (2)
**Frequency of potato consumption (8-level quasi-continuous)**						
	859 (28.7)	3.9 (1.9)	3 (2)	363 (28.3)	3.9 (2.0)	4 (2)
**Frequency of French-fries consumption (8-level quasi-continuous)**						
	859 (28.7)	3.4 (2.2)	3 (3)	363 (28.3)	3.3 (2.1)	3 (2)
**Frequency of vegetable consumption (8-level quasi-continuous)**						
	859 (28.7)	4.3 (2.1)	4 (3)	36 (28.3)	4.5 (2.2)	4 (3)
**Frequency of fruit and berry consumption (8-level quasi-continuous)**						
	859 (28.7)	3.5 (1.9)	3 (2)	363 (28.3)	3.5 (1.9)	3 (2)
**Frequency of wholegrain bread consumption (8-level quasi-continuous)**						
	859 (28.7)	3.0 (1.7)	3 (2)	363 (28.3)	2.9 (1.7)	3 (2)
**Frequency of fish consumption (8-level quasi-continuous)**						
	859 (28.7)	2.3 (1.1)	2 (1)	363 (28.3)	2.3 (1.1)	2 (1)
**Frequency of pizza consumption (8-level quasi-continuous)**						
	859 (28.7)	2.7 (1.1)	3 (1)	363 (23.3)	2.7 (1.0)	3 (1)
**Frequency of burger, kebab, or hotdog consumption (8-level quasi-continuous)**						
	859 (28.7)	2.3 (1.1)	2 (1)	363 (28.3)	2.3 (1.0)	2 (1)
**Frequency of candy consumption (8-level quasi-continuous)**						
	859 (28.7)	2.7 (1.1)	3 (1)	363 (28.3)	2.6 (1.1)	3 (1)
**Frequency of chocolate consumption (8-level quasi-continuous)**						

	859 (28.7)	2.4 (1.4)	2 (2)	363 (28.3)	2.4 (1.5)	2 (2)
**Frequency of chips consumption (8-level quasi-continuous)**						
	859 (28.7)	2.7 (1.6)	2 (2)	363 (28.3)	2.7 (1.6)	2 (2)
**Frequency of peanuts consumption (8-level quasi-continuous)**						
	240 (80.1)	1.8 (1.1)	1 (1)	113 (77.7)	1.8 (1.2)	1 (1)
**Frequency of fish oil consumption (8-level quasi-continuous)**						
	608 (49.5)	2.3 (1.2)	1 (2.5)	262 (48.2)	2.3 (1.9)	1 (2)
**Frequency of vitamin consumption (8-level quasi-continuous)**						
	608 (49.5)	2.8 (1.9)	2 (4)	262 (28.2)	2.8 (2.0)	2 (4)
**Frequency of physical activity/exercise (7-level quasi-continuous)**						
	859 (28.7)	4.9 (1.3)	5 (1)	363 (28.3)	5.0 (1.2)	5 (1)
**Hours of physical activity/exercise per week (6-level quasi-continuous)**						
	859 (28.7)	3.8 (1.4)	4 (2)	363 (28.3)	3.7 (1.3)	4 (2)
**Hours of TV per week (6-level quasi-continuous)**						
	859 (28.7)	3.2 (1.2)	3 (2)	363 (28.3)	3.2 (1.2)	3 (2)

¶ *i.e.* the average time spent in moderate to vigorous physical activity (MVPA) while wearing the monitor

Table S2 – Sample characteristics for binary and categorical variables

Variable	*Training set (n=1205)*		*Validation set (n=506)*	
	n **(% missing)**	**Frequency (%)**	***n*** **(% missing)**	**Frequency (%)**
**Gender (male)**				
	1199 (0.5)	585 (48.6)	503 (0.6)	243 (48.0)
**Master-level education for father**				
	780 (33.3)	261 (22.0)	314 (37.9)	77 (15.2)
**Master-level education for mother**				
	804 (33.3)	240 (19.9)	325 (35.8)	71 (14.0)
**Higher education for father (dichotomized)**				
	780 (35.3)	525 (67.3)	314 (37.4)	200 (63.7)
**Higher education for mother (dichotomized)**				
	804 (33.3)	580 (72.1)	325 (35.7)	233 (71.7)
**Norwegian food consumption**				
	230 (80.1)	214 (17.8)	101 (80.0)	95 (18.8)
**Food allergy**				
	230 (80.1)	32 (2.7)	101 (80.0)	13 (2.6)
**Attempting weight loss**				
	230 (80.1)	40 (3.3)	101 (80.0)	20 (4.0)
**Special diet**				
	230 (80.1)	24 (2.0)	101 (80.0)	5 (1.0)
**Vegetarian or vegan**				
	230 (80.9)	8 (0.7)	101 (80.0)	0 (0)
**Diabetic**				
	230 (80.9)	4 (0.3)	101 (80.0)	2 (0.4)
**Rating of diet (Categorical)**				
	230 (80.9)		101 (80.0)	
'Very healthy'				
		53 (23.0)		22 (21.8)
'Fairly healthy'				
		127 (55.2)		62 (61.4)
'Unhealthy'				
		3 (1.3)		2 (2.0)
'Don't know'				
		47 (20.4)		15 (14.9)
**Rating health and diet (Categorical)**				
	230 (80.9)		101 (80.0)	
**'No'**				
		12 (5.2)		13 (12.9)
'Not now'				
		12 (5.2)		5 (5.0)

'Yes'				
		149 (64.8)		63 (62.4)
'Don't know'				
		57 (24.8)		20 (19.8)
**Rating of weight (Categorical)**				
	230 (80.9)		101 (80.0)	
'OK'				
		206 (17.1)		89 (17.6)
'Too high'				
		15 (1.2)		8 (1.6)
'Too low'				
		9 (0.8)		4 (0.8)
**Reporting taking at least 2 hours of physical activity/exercise per week**				
	859 (28.7)	311 (36.2)	363 (28.2)	113 (31.1)

Table S3 - Crude unscaled effects of variables on Academic Performance and omitted variables (training set)¶

	ß (95% CI)	*n*	*P-value*	*R^2*
**Height (cm)**				
	0.15 (0.07 to 0.23)	*927*	*<.001****	*.02*
**Weight (kg)¶**				
	0.008 (-0.06 to 0.08)	*921*	*.80*	*<.001*
**Waist circumference (cm)**				
	-0.07 (-0.13 to -0.005)	*906*	*.04**	*.005*
**Systolic BP (mmHg)**				
	-0.025 (-0.07 to 0.02)	*885*	*.31*	*.001*
**Diastolic BP**				
	-0.006 (-0.08 to 0.06)	*879*	*.87*	*<.001*
**Right Hand strength (kg)¶**				
	0.19 (0.08 to 0.30)	*921*	*<.001****	*.01*
**Left Hand strength (kg)**				
	0.22 (0.11 to 0.33)	*903*	*<.001****	*.02*
**Average hand strength (kg)¶**				
	0.22 (0.11 to 0.33)	*901*	*<.001****	*.02*
**Muscle mass (kg)¶**				
	0.12 (-0.01 to 0.24)	*909*	*.07*	*.004*
**Body fat (%)**				
	-0.12 (-0.21 to -0.02)	*908*	*.02 ***	*.006*
**Fat mass (kg)¶**				
	-0.08 (-0.20 to 0.04)	*905*	*.18*	*.004*
**Fat free mass (kg)¶**				
	0.12 (0.00007 to 0.24)	*907*	*.05*	*.004*
**Bone mass (kg)**				
	2.47 (0.11 to 4.82)	*909*	*.04**	*.005*
**Protein mass (kg)¶**				
	0.55 (-0.002 to 1.11)	*891*	*.05*	*.004*
**Andersen aerobic fitness test (minutes)**				
	0.014 (0.010 to 0.019)	*857*	*<.001****	*.05*
**Stroop test congruent (ms)¶**				
	0.003 (-0.003 to -0.0018)	*817*	*<.001****	*.05*
**Stroop test incongruent (ms)**				
	-0.002 (-0.0026 to -0.0014)	*817*	*<.001****	*.05*
**Stroop control (ms)¶**				
	-0.002 (-0.003 to -0.002)	*817*	*<.001****	*.04*
**Sedentary exercise (minutes)**				
	0.00006 (-0.00058 to 0.0007)	*833*	*.86*	*<.001*
**Light exercise (minutes)**				
	-0.002 (-0.003 to -0.0006)	*833*	*.006***	*.008*
**Moderate exercise (minutes)**				
	-0.0002 (-0.004 to 0.003)	*833*	*.93*	*<.001*
**Hard exercise (minutes) ¶**				

	0.01 (0.004 to 0.02)	*827*	*.009***	*.008*
**Percentage of sedentary activity¶**				
	0.10 (0.004 to 0.20)	*833*	*.04**	*.005*
**Percentage of light activity**				
	-0.32 (-0.48 to -0.17)	*833*	*<.001****	*.02*
**Percentage of moderate activity¶**				
	0.05 (-0.17 to 0.28)	*833*	*.64*	*<.001*
**Percentage of hard activity**				
	0.87 (0.29 to 1.46)	*827*	*.003***	*.01*
**Total Moderate to vigorous physical activity (MVPA) (cpm)¶**				
	0.001 (-0.002 to 0.004)	*838*	*.49*	*.001*
**Average MVPA (cpm)¶**				
	0.01 (-0.01 to 0.04)	*838*	*.19*	*.002*
**Average steps (cpm)¶**				
	1.08 (0.04 to 2.13)	*837*	*.04**	*.005*
**Child-rated QoL (LQ100)**				
	0.11 (0.06 to 0.16)	*894*	*.003***	*.02*
**Parent-rated child QoL (LQ100)**				
	0.02 (0.11 to 0.24)	*597*	*<.001****	*.005*
**Effect of master-level education for father**				
	5.93 (4.62 to 7.23)	*636*	*<.001****	*.11*
**Effect of master-level education for mother**				
	5.90 (4.57 to 7.22)	*654*	*<.001****	*.11*
**Effect of being a boy**				
	-0.39 (-1.46 to 0.67)	*960*	*.47*	*<.001*
**Effect of father having higher education**				
	4.75 (3.39 to 6.11)	*636*	*<.001****	*.07*
**Effect of mother having higher education**				
	4.73 (3.31 to 6.14)	*654*	*<.001****	*.06*
**Frequency of eating lunch each week (quasi-continuous)**				
	-0.10 (-0.38 to 0.19)	*681*	*.49*	*<.001*
**Frequency of eating dinner each week (quasi-continuous)**				
	0.33 (-0.06 to 0.72)	*681*	*.10*	*.004*
**Frequency of eating supper each week (quasi-continuous)**				
	-0.13 (-0.33 to 0.08)	*681*	*.23*	*.002*
**Frequency of eating packed lunch (quasi-continuous)**				
	-0.23 (-0.50 to 0.04)	*681*	*.09*	*.004*
**Frequency of whole milk consumption (quasi-continuous)**				
	-0.28 (-0.65 to 0.09)	*681*	*.14*	*.003*

**Frequency of low-fat milk consumption (quasi-continuous)**				
	-0.14 (-0.53 to 0.24)	*681*	*.46*	*<.001*
**Frequency of extra-low-fat milk consumption (quasi-continuous)**				
	-0.13 (-0.52 to 0.25)	*681*	*.50*	*<.001*
**Frequency of skimmed milk consumption (quasi-continuous)**				
	-0.62 (-1.14 to -0.10)	*681*	*.02**	*.008*
**Frequency of orange juice consumption (quasi-continuous)**				
	-0.36 (-0.81 to 0.09)	*681*	*.12*	*.004*
**Frequency of sugared cordial consumption (quasi-continuous)**				
	-0.73 (-1.26 to -0.19)	*681*	*.008***	*.01*
**Frequency of sugar-free cordial consumption (quasi-continuous)**				
	-0.72 (-1.27 to 0.17)	*681*	*.01**	*.01*
**Frequency of sugared soda consumption (quasi-continuous)**				
	-0.036 (-0.95 to 0.24)	*681*	*.24*	*.002*
**Frequency of potato consumption (quasi-continuous)**				
	0.10 (-0.22 to 0.43)	*681*	*.53*	*<.001*
**Frequency of French-fries consumption (quasi-continuous)**				
	-0.10 (-0.39 to 0.19)	*681*	*.51*	*<.001*
**Frequency of vegetable consumption (quasi-continuous)**				
	0.14 (-0.16 to 0.45)	*681*	*.35*	*.001*
**Frequency of fruit and berry consumption (quasi-continuous)**				
	0.23 (-0.11 to 0.58)	*681*	*.18*	*.003*
**Frequency of wholegrain bread consumption (quasi-continuous)**				
	0.10 (-0.25 to 0.45)	*681*	*.57*	*<.001*
**Frequency of fish consumption (quasi-continuous)**				
	-0.34 (-0.94 to 0.26)	*681*	*.26*	*.002*
**Frequency of pizza consumption (quasi-continuous)**				
	-0.95 (-1.56 to -0.34)	*681*	*.002***	*.01*

**Frequency of burger, kebab, or hotdog consumption (quasi-continuous)**				
	-0.60 (-1.20 to -0.0002)	*681*	*.05*	*.006*
**Frequency of candy consumption (quasi-continuous)**				
	-1.1 (-1.64 to -0.56)	*681*	*<.001****	*.02*
**Frequency of chocolate consumption (quasi-continuous)**				
	-0.01 (-0.47 to 0.44)	*681*	*.95*	*<.001*
**Frequency of chips consumption (quasi-continuous)**				
	0.02 (-0.37 to 0.41)	*681*	*.92*	*<.001*
**Frequency of peanuts consumption (quasi-continuous)**				
	-0.30 (-1.48 to 0.89)	*182*	*.62*	*.001*
**Frequency of fish oil consumption (quasi-continuous)**				
	-0.04 (-0.43 to 0.35)	*482*	*.84*	*<.001*
**Frequency of vitamin consumption (quasi-continuous)**				
	0.19 (-0.19 to 0.57)	*482*	*.33*	*.002*
**Effect of reporting Norwegian food consumption**				
	2.27 (-3.40 to 7.93)	*174*	*.43*	*.004*
**Effect of reporting food allergy**				
	-0.42 (-4.06 to 3.23)	*174*	*.82*	*<.001*
**Effect of reporting weight loss**				
	-2.03 (-5.26 to 1.20)	*174*	*.22*	*.01*
**Effect of reporting special diet**				
	-2.70 (-7.03 to 1.63)	*174*	*.22*	*.01*
**Effects of rating of diet (Categorical)**				
		174		*.10*
'Fairly healthy'				
	1.09 (-1.85 to 4.02)	-	*.15*	-
'Unhealthy'				
	-12.43 (-23.90 to -0.96)	-	*.034**	-
'Don't know'				
	-4.89 (-8.55 to -1.23)	-	*.009***	-
**Effects of rating of health and diet (Categorical)**				
		174		*.04*
'Not now'				
	-5.85 (-13.78 to 2.08)	-	*.15*	-
'Yes'				
	-0.68 (-6.65 to 5.28)	-	*.82*	-

'Don't know'				
	-4.07 (-10.37 to 2.22)	*-*	*.20*	*-*
**Effects of rating of weight (Categorical)**				
		174		*.01*
'Too high'				
	0.77 (-4.19 to 5.73)	*-*	*.76*	*-*
'Too low'				
	-4.59 (-11.47 to 2.30)	*-*	*.19*	*-*
**Frequency of physical activity/exercise (quasi-continuous)**				
	0.94 (0.46 to 1.41)	*681*	*<.001****	*.02*
**Hours of physical activity/exercise per week (quasi-continuous)**				
	1.29 (0.83 to 1.74)	*681*	*<.001*	*.04*
**Hours of TV per week (quasi-continuous)**				
	0.38 (-0.13 to 0.89)	*681*	*.14*	*.04*

* *P*<0.05, ***P*<0.01, ****P*<0.001

¶ Omitted from modelling due to collinearity with other predictor variables

Table S4 – Variable selections of the machine learning techniques

Algorithm	Selected variables for AP set	Selected variables for QoL set
Random forest	Fitness test; frequency of candy consumption; frequency of eating supper each week; mother having master's degree; rating of weight; frequency of sugared soda consumption.	Percentage of time in light exercise; average national test score; fitness test; LQ0100 Parent; gender.
Random forest (sensitivity)	Percentage of time in light exercise; attempting weight loss; Father having master's degree; frequency of potato consumption; rating of own diet.	Hard exercise; percentage of time in light exercise; LQ0100 Parent; frequency of physical activity; gender.
Support vector machine	Mother having master's degree; frequency of peanut consumption; Stroop incongruent test score; frequency of dinner consumption; rating of own diet; frequency of fruit and berry consumption; frequency of candy consumption; frequency of supper consumption; frequency of physical exercise; own weight assessment.	LQ0100 Parent; fitness test; average national test score.
Support vector machine (sensitivity)	Mother having master's degree; attempting weight loss; fitness test; sedentary exercise; rating of own diet; light exercise; Stroop incongruent test score; low fat milk consumption; percentage of time in light exercise.	LQ0100 Parent; frequency of physical exercise; fitness test; frequency of orange juice consumption.
k Nearest neighbors	Mother having higher education; own weight assessment.	LQ0100 Parent; fish oil consumption; father having master's degree; frequency of chips consumption; frequency of French fries consumption.
k Nearest neighbors (sensitivity)	Special diet status; Father having master's degree; rating of own diet.	LQ0100 Parent; frequency of fish consumption; frequency of lunch consumption; average national test score; frequency of physical exercise; hours of watching TV or using computer.
Neural net	Mother having master's degree; frequency of peanut consumption; Stroop incongruent test score; mother having higher education.	LQ0100 Parent; frequency of fish consumption; frequency of pizza consumption.

Neural net (Sensitivity)	Father having master's degree; hours of physical activity/exercise; diabetes status.	LQ0100 Parent; fitness test.

Table S5 - Crude unscaled effects of variables on child-rated quality of life (LQ-100) and omitted variables (training set)¶

	ß (95% CI)	***n***	***P-value***	***R^2***
**Height (cm)**				
	0.006 (-0.08 to 0.09)	*1,105*	*.89*	*<.001*
**Weight (kg)¶**				
	-0.01 (-0.09 to 0.06)	*1,098*	*.69*	*<.001*
**Waist circumference (cm)**				
	-0.05 (-0.12 to 0.12)	*1,086*	*.11*	*.002*
**Systolic BP (mmHg)**				
	0.01 (-0.04 to 0.06)	*1,064*	*.68*	*<.001*
**Diastolic BP**				
	-0.01 (-0.09 to 0.06)	*1,058*	*.70*	*<.001*
**Right Hand strength (kg)¶**				
	0.03 (-0.08 to 0.14)	*1,091*	*.68*	*<.001*
**Left Hand strength (kg)**				
	0.04 (-0.07 to 0.15)	*1,089*	*.49*	*<.001*
**Average hand strength (kg)¶**				
	0.04 (-0.8 to 0.15)	*1,087*	*.62*	*<.001*
**Muscle mass (kg)¶**				
	0.01 (-0.13 to 0.14)	*1,092*	*.91*	*<.001*
**Body fat (%)**				
	-0.04 (-0.15 to 0.06)	*1,092*	*.42*	*<.001*
**Fat mass (kg)¶**				
	0.02 (-0.11 to 0.16)	*1,089*	*.74*	*<.001*
**Fat free mass (kg))¶**				
	-0.01 (-0.13 to 0.12)	*1,091*	*.90*	*<.001*
**Bone mass (kg)**				
	0.08 (-2.44 to 2.61)	*1,093*	*.95*	*<.001*
**Protein mass (kg))¶**				
	-0.01 (-0.60 to 0.58)	*1,073*	*.97*	*<.001*
**Fitness test (minutes)**				
	0.01 (0.001 to 0.02)	*1,008*	*<.001****	*.03*
**Stroop test congruent (ms)¶**				
	-0.0002 (-0.001 to 0.0006)	*982*	*.62*	*<.001*
**Stroop test incongruent (ms)**				
	-0.00007 (-0.0007 to 0.0006)	*982*	*.83*	*<.001*
**Stroop control (ms)¶**				
	0.0003 (-0.0005 to 0.001)	*982*	*.44*	*<.001*
**Sedentary exercise (minutes)**				

	<0.001 (<-0.001 to 0.001)	*971*	*.99*	*<.001*
**Light exercise (minutes)**				
	0.001 (<0.001 to 0.003)	*971*	*.12*	*.002*
**Moderate exercise (minutes)¶**				
	0.007 (0.003 to 0.01)	*971*	*<.001****	*.01*
**Hard exercise (minutes)**				
	0.03 (0.02 to 0.04)	*964*	*<.001****	*.03*
**Percentage of time sedentary ¶**				
	-0.16 (-0.27 to -0.05)	*971*	*.003***	*.01*
**Percentage of time in light exercise**				
	0.07 (-0.10 to 0.23)	*971*	*.41*	*<.001*
**Percentage of time in moderate exercise¶**				
	0.46 (0.21 to 0.70)	*971*	*<.001****	*.01*
**Percentage of time in vigorous exercise**				
	1.44 (0.86 to 2.03)	*964*	*<.001****	*.02*
**Total moderate to vigorous physical activity (MVPA) (cpm)¶**				
	0.01 (0.004 to 0.01)	*974*	*<.001****	*.02*
**Average MVPA (cpm)**				
	0.06 (0.04 to 0.08)	*974*	*<.001****	*.03*
**Average steps**				
	3.03 (1.89 to 4.17)	*974*	*<.001****	*.03*
**Average national test score**				
	0.17 (0.09 to 0.25)	*894*	*<.001****	*.02*
**Parent-rated child QoL (LQ100)**				
	0.25 (0.19 to 0.31)	*663*	*<.001****	*.08*
**Effect of master-level education for father¶**				
	2.35 (0.77 to 3.94)	*732*	*.004***	*.01*
**Effect of master-level education for mother¶**				
	0.99 (-0.62 to 2.61)	*732*	*.23*	*<.001*
**Effect of being a boy**				
	0.33 (-0.90 to 1.56)	*1,107*	*.60*	0.0002
**Effect of father having higher education**				
	2.56 (0.97 to 4.15)	*732*	*.002***	*.01*
**Effect of mother having higher education**				
	1.94 (0.30 to 3.58)	*756*	*.02***	*.007*
**Frequency of eating lunch each week (quasi-continuous)**				
	0.47 (0.15 to 0.80)	*787*	*.004***	*.009*
**Frequency of eating dinner each week (quasi-continuous)**				
	0.26 (-0.17 to 0.68)	*787*	*.24*	*.002*
**Frequency of eating supper each week (quasi-continuous)**				

	0.05 (-0.19 to 0.28)	*787*	*.70*	*<.001*
**Frequency of eating packed lunch (quasi-continuous)**				
	-0.05 (-0.34 to 0.27)	*787*	*.77*	*<.001*
**Frequency of whole milk consumption (quasi-continuous)**				
	-0.22 (-0.64 to 0.19)	*787*	*.29*	*<.001*
**Frequency of low-fat milk consumption (quasi-continuous)**				
	-0.13 (-0.31 to 0.57)	*787*	*.56*	*<.001*
**Frequency of extra-low-fat milk consumption (quasi-continuous)**				
	0.11 (-0.34 to 0.57)	*787*	*.63*	*<.001*
**Frequency of skimmed milk consumption (quasi-continuous)**				
	-0.55 (-1.14 to 0.04)	*787*	*.07*	*.003*
**Frequency of orange juice consumption (quasi-continuous)**				
	0.57 (0.05 to 1.08)	*787*	*.03**	*.006*
**Frequency of sugared cordial consumption (quasi-continuous)**				
	-0.58 (-1.19 to 0.03)	*787*	*.06*	*.004*
**Frequency of sugar-free cordial consumption (quasi-continuous)**				
	0.50 (-0.12 to 1.12)	*787*	*.11*	*.002*
**Frequency of sugared soda consumption (quasi-continuous)**				
	-0.01 (-0.70 to 0.67)	*787*	*.97*	*<.001*
**Frequency of potato consumption (quasi-continuous)**				
	0.30 (-0.08 to 0.67)	*787*	*.12*	*.003*
**Frequency of French-fries consumption (quasi-continuous)**				
	0.21 (-0.12 to 0.54)	*787*	*.22*	*.002*
**Frequency of vegetable consumption (quasi-continuous)**				
	0.32 (-0.02 to 0.66)	*787*	*.06*	*.003*
**Frequency of fruit and berry consumption (quasi-continuous)**				
	0.43 (0.05 to 0.82)	*787*	*.03**	*.005*
**Frequency of wholegrain bread consumption (quasi-continuous)**				
	0.14 (-0.28 to 0.57)	*787*	*.50*	*<.001*

**Frequency of fish consumption (quasi-continuous)**				
	0.34 (-0.33 to 1.01)	*787*	*.32*	*.001*
**Frequency of pizza consumption (quasi-continuous)**				
	0.07 (-0.62 to 0.76)	*787*	*.85*	*<.001*
**Frequency of burger, kebab, or hotdog consumption (quasi-continuous)**				
	-0.23 (-0.91 to 0.45)	*787*	*.50*	*<.001*
**Frequency of candy consumption (quasi-continuous)**				
	-0.29 (-0.94 to 0.36)	*787*	*.39*	*.001*
**Frequency of chocolate consumption (quasi-continuous)**				
	0.12 (-0.34 to 0.63)	*787*	*.63*	*<.001*
**Frequency of chips consumption (quasi-continuous)**				
	0.06 (-0.39 to 0.51)	*787*	*.80*	*<.001*
**Frequency of peanuts consumption (quasi-continuous)**				
	-1.12 (-2.35 to 0.11)	*216*	*.07*	*<.001*
**Frequency of fish oil consumption (quasi-continuous)**				
	0.55 (0.07 to 1.01)	*552*	*.02**	*.008*
**Frequency of vitamin consumption (quasi-continuous)**				
	0.07 (-0.39 to 0.54)	*552*	*.75*	*<.001*
**Effect of reporting Norwegian food consumption**				
	-1.52 (-6.79 to 3.76)	*219*	*.57*	*.002*
**Effect of reporting food allergy**				
	-2.54 (-6.29 to 1.28)	*219*	*.18*	*.008*
**Effect of reporting attempting weight loss**				
	-2.70 (-6.12 to 0.73)	*219*	*.12*	*.01*
**Effect of reporting special diet**				
	-3.27 (-7.74 to 1.01)	*219*	*.13*	*.01*
**Effects of rating of diet (Categorical)**				
		219		*.09*
'Fairly healthy'				
	-3.42 (-6.49 to -0.35)	-	*.03**	-
'Unhealthy'				
	-16.75 (-27.66 to -5.84)	-	*.003***	-
'Don't know'				

	-7.28 (-11.06 to -3.5)	*-*	*<.001****	*-*
**Effects of rating of health and diet (Categorical)**				
		219		*.01*
'Not now'				
	-4.73 (-12.72 to 3.24)	*-*	*.24*	*-*
'Yes'				
	-0.91 (-6.66 to 4.82)	*-*	*.75*	*-*
'Don't know'				
	-2.39 (-12.71 to 3.24)	*-*	*.44*	*-*
**Effects of rating of weight (Categorical)**				
		219		*.03*
'Too high'				
	-7.62 (-12.66 to -2.59)	*-*	*.003**	*-*
'Too low'				
	-1.23 (-8.01 to 5.54)	*-*	*.72*	*-*
**Frequency of physical activity/exercise (quasi-continuous)**				
	1.42 (0.89 to 1.95)	*787*	*<.001****	*.03*
**Hours of physical activity/exercise per week (quasi-continuous)**				
	1.10 (0.58 to 1.61)	*787*	*<.001****	*.02*
**Hours of TV per week (quasi-continuous)**				
	-1.10 (-1.68 to -0.52)	*787*	*<.001****	*.02*

**P<0.05, **P<0.01, ***P<0.001*

¶ Omitted from modelling due to collinearity with other predictor variables

Table S6 – adjusted effects for fitted mixed Regression Model 1 for predicting quality of life

	ß (95% CI)	*n*	*P-value*
		293	
**Hard exercise (minutes)**			
	0.02 (0.0007 to 0.04)	*-*	*.04**
**Fish oil consumption (dichotomised)**			
	2.09 (-0.09 to 4.26)	*-*	*.06*
**Parent-rated child QoL (LQ100)**			
	0.25 (0.16 to 0.35)	*-*	*<.001****

Notes

Note 1: For all models apart from the hierarchical model, we calculated adjusted R^2 as 1-$(1 - R^2) \cdot \left(\frac{n-1}{(n-(k+1))}\right)$; where n is sample size and k is the number of explanatory variables in the model. For the mixed model, in which the appropriateness of adjusted R^2 is debated and there is no accepted practice, we predicted residuals for the fixed part of the model only, and reported the square of the residual correlation with the dependent variable. To check adequacy of fit in regression models, we examined residuals graphically.

Note 2: The AP regression model for the sensitivity analysis with imputed data included the following variables: left hand strength, master education for mother and father, fitness test, light exercise, child and parent LQ0100 scores, higher education for father, reported hours of physical activity/exercise, reported packed lunch frequency, skimmed milk consumption frequency, frequency of pizza consumption, and reporting of a special diet.

Note 3: The QoL regression model for the sensitivity analysis with imputed data included the following variables: fitness test, reported frequency of orange juice consumption, hard exercise, parent LQ0100 score, reported hours of physical activity/exercise, reported lunch frequency, and reported frequency of TV and computer use.

Note 4: Adjusted for other variables in the model, each increase in quasi-continuous physical activity/exercise category (ie*,* through categories 'never, to <1 per month, 1 to 3 per month, 1 per week, 2 to 3 per week, 4 to 6 per week, to every day' sessions of exercise that cause sweat and being out of breath) is associated with a 1.1 unit increase in QoL. To aid interpretation, the difference between taking no exercise, and exercising once per week, equates approximately to a small-to-medium effect size (ie*,* Cohen's d=0.32). Each reported increase in quasi-continuous TV or computer use category (ie*,* through categories 'none, to <0.5, 0.5 to 1, 2 to 3, 4, or over 4 hours per day') is associated with a -0.95 unit decrease in QoL (ie*,* going from 0 to 2 hours per day equates to a small-to-medium effect; d=-0.28). An increase of 100 minutes of recorded hard exercise equates to a small effect (d=0.19) and a 10% increase in moderate exercise equates to a small-to-medium (d=0.28) increase in QoL. There was some evidence (P=.02) of association between AP and QoL after adjustment for the model variables; a 20 unit increase AP is associated with a 2.24 unit increase in QoL (which equates to a small effect (d=0.22)). From Regression Model 1, there is very strong evidence that parental assessment of child QoL is associated with child QoL assessment after adjustment for hard exercise and fish-oil consumption; a ten unit increase in parental assessment of QoL being associated with a 2.5 unit increase in child-rated quality of life (which equates to a small effect (d=0.22)).

Adjusted for parental education, TV or computer use, Stroop cognitive function, and hand strength, there is very strong evidence that reporting taking exercise that causes a sweat, for at least 2 hours per week, is associated with a 2.5 unit increase in AP (ie*,* a small-to-medium effect; d=0.29). There is very strong evidence that hand strength is associated with a small increase in AP; a 10kg increase equating to a small effect (d=0.24). There is some evidence (ie*,* P<.05) of a mother having university education equating to a small increase in AP; d=0.21), very strong evidence (nb*,* even after adjustment for university education) that a mother having master's-level education or above equates to a further small effect; d=0.23), and weak evidence (P=.06) that a father having master's-level or above equates to a small effect on AP (d=0.19). There is some evidence that each reported increase in TV or computer

use category is associated with a 1.2 unit increase in AP (eg, going from ‘never’ to 2 hours per day equates to a medium effect; *d*=0.42). Finally, there is very strong evidence that congruent Stroop test score is associated with AP, with a reduction of 1 second being associated with an increase of 3.7 AP units (ie*,* a medium effect; *d*=0.44).
